# Image-based modeling of vascular organization to evaluate anti-angiogenic therapy

**DOI:** 10.1186/s13062-023-00365-x

**Published:** 2023-03-15

**Authors:** David Ascheid, Magdalena Baumann, Caroline Funke, Julia Volz, Jürgen Pinnecker, Mike Friedrich, Marie Höhn, Rajender Nandigama, Süleyman Ergün, Bernhard Nieswandt, Katrin G. Heinze, Erik Henke

**Affiliations:** 1 Institute of Anatomy and Cell Biology, Universität Würzburg, Koellikerstrasse 6, 97070 Würzburg, Germany; 2 Institute of Experimental Biomedicine I, Universitätsklinikum Würzburg, Würzburg, Germany; 3 Rudolf Virchow Center for Integrative and Translational Bioimaging, Universität Würzburg, Josef-Schneider-Straße 2, 97080 Würzburg, Germany; 4 Graduate School for Life Sciences, Universität Würzburg, Würzburg, Germany

**Keywords:** Vascular structure, Cancer, Tumor microenvironment, Optical clearing, Light sheet fluorescence microscopy, 3D image analysis

## Abstract

In tumor therapy anti-angiogenic approaches have the potential to increase the efficacy of a wide variety of subsequently or co-administered agents, possibly by improving or normalizing the defective tumor vasculature. Successful implementation of the concept of vascular normalization under anti-angiogenic therapy, however, mandates a detailed understanding of key characteristics and a respective scoring metric that defines an improved vasculature and thus a successful attempt. Here, we show that beyond commonly used parameters such as vessel patency and maturation, anti-angiogenic approaches largely benefit if the complex vascular network with its vessel interconnections is both qualitatively and quantitatively assessed. To gain such deeper insight the organization of vascular networks, we introduce a multi-parametric evaluation of high-resolution angiographic images based on light-sheet fluorescence microscopy images of tumors. We first could pinpoint key correlations between vessel length, straightness and diameter to describe the regular, functional and organized structure observed under physiological conditions. We found that vascular networks from experimental tumors diverted from those in healthy organs, demonstrating the dysfunctionality of the tumor vasculature not only on the level of the individual vessel but also in terms of inadequate organization into larger structures. These parameters proofed effective in scoring the degree of disorganization in different tumor entities, and more importantly in grading a potential reversal under treatment with therapeutic agents. The presented vascular network analysis will support vascular normalization assessment and future optimization of anti-angiogenic therapy.

**Supplementary Information:**

The online version contains supplementary material available at 10.1186/s13062-023-00365-x.

## Introduction

A functional, mature and organized vascular network is essential for adequate supply and tissue homeostasis. To accommodate for the diverse metabolic needs and organ specific functions architecture of the vasculature varies strongly between different organs. However, there are also general determinants for an effective and economic formation of the vasculature. These fundamental factors lead in all organs to a vessel architecture that can be described as regular and organized. Under pathological conditions like tumor formation, fibrosis and inflammation, the functionality and the organization of the vasculature can be significantly compromised, and divert from a regular design (reviewed in [1–3]). In consequence, supply and drug transport into the affected tissues are heterogeneous and often insufficient.

In the context of the defective and dysfunctional tumor vasculature the concept of “vascular normalization*”* has been established. The term was coined to describe the vascular changes observed in tumors after treatment with anti-angiogenic drugs [4]. As the defectiveness of the tumor vasculature is caused by an abundance of pro-angiogenic factors, the reasoning was that a blockage of the angiogenic signals should revert or *normalize* the pathological vascular phenotype. This should positively affect drug transport, treatment efficacy and malignant behavior [5–8]. Indeed, treatment with various anti-angiogenic drugs can improve vascular support by pericytes and reduce permeability [9–11]. These characteristics are often used as surrogate markers for the effectiveness of anti-angiogenic therapy and because they are experimentally effortlessly accessible studies rely on them to demonstrate reduction of vascular dysfunctionality. However, these characteristics are only a measure for vascular maturation and patency and do not allow a statement about the vasculature’s ability to provide adequate supply. In fact, it has been shown that despite improving vessel maturation and patency, anti-angiogenic therapy reduces in many settings transport of nutrients, oxygen and drugs into tumors [12–15].

Independently of the still open question, if anti-angiogenic drugs are able to improve drug transport or if other vascular-targeted approaches have to be employed, the concept of “vascular normalization” promises the possibility to strategically alter the vasculature by aiming to improve drug transport and treatment efficacy [16–18]. For such a target-oriented approach, we still lack an understanding what defines a “normal”—or better an efficient—vascular network. In particular we need to know which experimentally accessible and quantifiable parameters indicate efficacy of the vascular network. This would allow defining a therapeutic goal for vascular-targeted therapy. Moreover, this basic understanding about vascular organization and supply efficacy would also impact other research fields: e.g. the current goal in tissue engineering is the generation of pre-vascularized ready-to-implant grafts [19]. A well-organized vasculature in the implants would increase rates of successful engraftment and reduce the amount of graft tissue needed, as less of the engrafted material will succumb to necrosis [20–23].

We aimed to develop a quantitative model describing vascular organization. By comparing results derived from this model in normal organs and in tumors, we identified key parameters that distinguish effective from ineffective vascular networks. These parameters allowed accessing and rating the effect of anti-angiogenic therapies or other vascular-targeted approaches towards a more effective structure of the tumor vasculature.

## Results

For a detailed analysis of capillary networks, we performed light-sheet fluorescence microscopy (LSFM) on optically cleared murine organs and tumors. Optical clearing of the otherwise opaque sample is a prerequisite and often a challenge in LSFM. The two most frequently used methods (reviewed in [24]) are based on different concepts: solvent-based clearing replaces the tissues’ water with an organic solvent of high refractive index close to that of the remaining dehydrated proteins. In contrast, hyperhydration methods aim to reduce the tissues’ refractive index.

We tested one hyperhydration technique (CUBIC [25]) and two solvent-based methods (iDISCO [26] and an ethyl cinnamate (EtCi)-based protocol [27]) for their suitability to prepare pre-stained tissue samples when imaged by LSFM (conditions are listed in Additional file 1: Table S1). In addition, we evaluated several methods to decolorize tissue, i.e. by removing heme from the samples that is known for its autofluorescence: This included treatment with (i) hydrogen peroxide, (ii) the CUBIC1 formulation, and (iii) with 25% Quadrol (*N,N,N*′*,N*′-tetrakis-(2-hydroxypropyl)ethylenediamine), the heme-removing agent in CUBIC1 [28]. All treatment removed heme completely (Additional file 1: Figure S1A). While the decolorization with Quadrol took considerably longer, the procedure, unlike hydrogen peroxide treatment, preserved fluorescent signal from pre-staining within the tissue (Additional file 1: Figure S2A). The two methods therefore can supplement each other. The more laborious to prepare and viscous CUBIC1 solution had no apparent benefits over 25% Quadrol alone.

Like all hyperhydration protocols CUBIC is time consuming, and thus a clear disadvantage compared to solvent-based methods. Clearing results with the two solvent-based methods were comparable (Additional file 1: Figure S1B). However, the last steps of the iDISCO procedure left most tissues brittle, which can cause problems in handling of the tissue blocks, especially, depending on the mounting mechanism during introduction of the blocks into the optical chamber of the LSF-microscope (BABB-based clearing [29] resulted in similar problems as iDISCO). In combination with the higher price and toxicity of the iDISCO reagents, we favored the EtCi method and used it exclusively in the following experiments. EtCi-clearing is compatible with immuno fluorescence staining, it works on all tested organs and we found excellent preservation of the fluorescent signal for at least 12 months. An often-cited disadvantage of solvent-based clearing is the shrinking of the cleared tissue [24]. We found it to be acceptable, at 6.3 ± 2.1% in each direction for EtCi (Additional file 1: Figure S2B). CUBIC on the other hand also results in changed morphology, as the tissue expands during clearing (Additional file 1: Figure S1B, S2C) [24]. Based on these observations, we developed a workflow to determine the optimal steps, depending on the experimental aims (Additional file 1: Figure S2D).

### Descriptive analysis of 3D-angiography

After having established a suitable clearing and imaging method, we next evaluated methods for intravital staining and parametrization of imaging results. C57Bl/6Jmice were injected i.v. with fluorescent-labeled antibodies recognizing various endothelial markers. Organs were harvested and subjected to the described fixation and clearing procedure. The antibody recognizing CD105 (endoglin) yielded consistent and reproducible results in the tested organs. Antibodies directed against other established endothelial surface antigens resulted in some organs in weak signals or did not stain the entire vasculature. These results indicated an organ-specific expression of the respective antigens or expression regulation coupled to the functional state or size of the individual vessels.

**Fig. 1:**
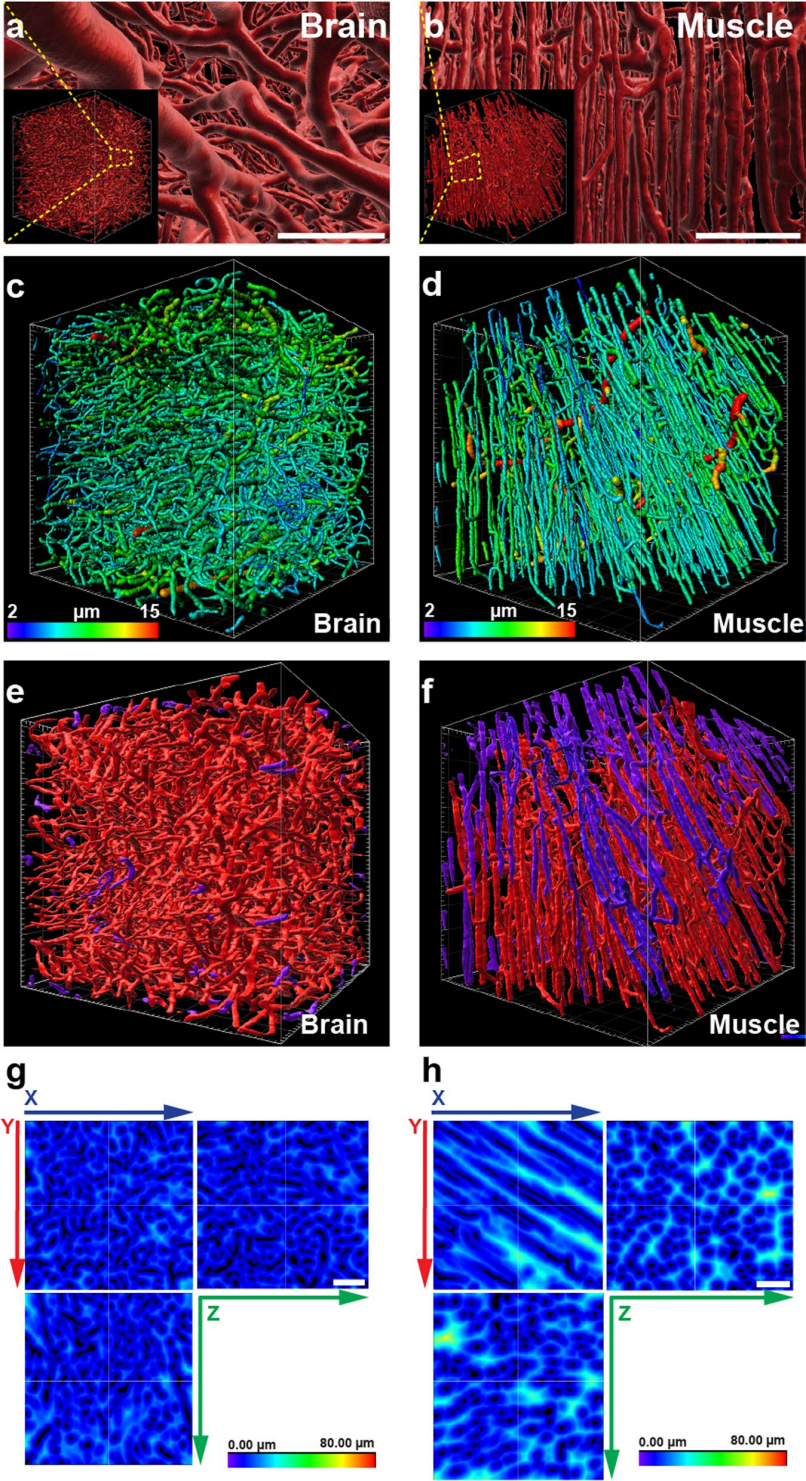
Vascular structures in healthy organs. **a** Representative 3D-projection of the perfused vasculature in the frontal cortex of the murine brain after segmentation. SB: 100 µm. Insert: full representation of the tissue fragment acquired by LSFM. Grid scale: 50 µm. **b** Representative 3D-projection of the perfused vasculature in the murine skeletal muscle after segmentation. SB: 100 µm. Insert: full representation of the tissue fragment acquired by LSFM. Grid scale: 50 µm. **c** Tracing results of perfused vessels in the murine brain. Vessel segments are displayed color-coded according to average diameters. Grid scale: 50 µm; Color scale: Average vessel segment diameter: 2-15 µm. **d** Tracing results of perfused vessels in the murine skeletal muscle. Vessel segments are displayed color-coded according to average diameters. Grid scale: 50 µm; Color scale: Average vessel segment diameter: 2-15 µm. **e** Rendering of segmented vasculature in the murine brain. The majority of vessels in the displayed cubic section forms an interconnected structure (red). On the surfaces of the displayed structure are few vessel segments (purple) observable that are not connected within the visible volume to the bulk of vessels. Grid scale: 50 µm. **f** Rendering of segmented vasculature in the murine skeletal muscle. The majority of vessels in the displayed cubic section forms an interconnected structure (red). On the surfaces of the displayed structure several large vessel segments (purple) are observable that are not connected within the visible volume to the bulk of vessels. Grid scale: 50 µm. **g** Heatmap display of distances from nearest perfused vessel in the murine brain. SB: 100 µm. **h** Heatmap display of distances from nearest perfused vessel in the murine skeletal muscle. SB: 100 µm

In our effort to find experimentally accessible parameters for vascular structures, we focused on four organs that displayed uniformly stained vessel networks within the field of view (FOV): brain (frontal lobe), retina (i.e. the vessels of the *choroidea*), skeletal muscle (*M. vastus lateralis*) and the mucosa of the colon (Fig. 1A,B and Additional file 1: Fig S3A,B). The four selected organs provided a significant variety of vessel density and network complexity. Other organs were excluded from detailed analysis for various reasons: the myocard’s vasculature showed a similar structure as the skeletal muscles, although with higher vessel density (Additional file 1: Fig S4A). Likewise, the stomach’s vasculature is similar to the colon’s vasculature (Additional file 1: Fig S4B). Consequently, both samples of the myocard and the stomach were omitted in order to not perturb downstream analysis by redundancy of the selected probes. In the liver, the discontinuous sinusoidal endothelium prohibited successful reconstruction, while in the renal cortex the strongly stained glomeruli impeded analysis (Additional file 1: Fig S4C,D).

**Fig. 2:**
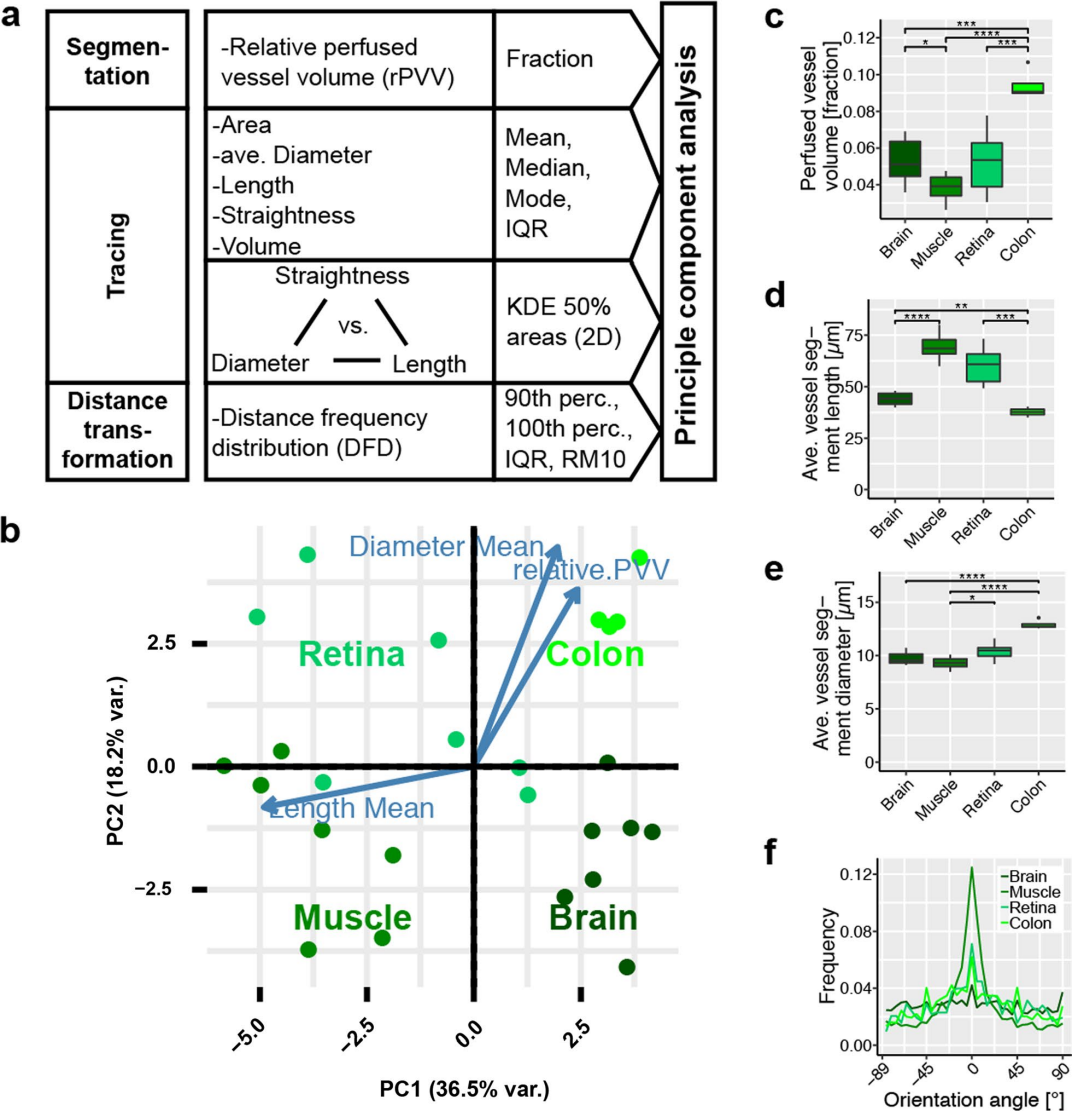
Evaluation process and statistical analysis: Results for healthy organs. **a** Schematic representation of the approach for determine variables used in principal component analysis (PCA). **b** PCA for the healthy tissues. Orientations of the three highest ranked variables are shown as blue arrows. **c** Relative perfused vessel volume (rPVV) as fraction of the whole tissue volume. n ≥ 6. **d** Average vessel segment length in selected organs. n ≥ 6. **e** Average vessel segment diameter in selected organs. n ≥ 6. **f** Vessel orientation angle within the XY-plane in selected organs. Angles centered around most frequent orientation at 0°. n ≥ 6, for clarity only the average values are shown. Error bars: ± SEM. Asterisks display results from statistical tests with *: P < 0.05, **: P < 0.01, ***: P < 0.001, ****: P < 0.0001

In the selected four organs, the vascular networks were reconstructed using the Imaris software. Additionally, to get a detailed description of the vasculature in these organs, including diameters, orientation and connection status of individual vessel segments, a tracing routine was established (Fig. 1C,D and Additional file 1: Fig S3C,D). Importantly, by providing a compressive report of how individual segments are connected to each other the tracing routine delivered a numeric description of the vessel networks architecture in its entirety (Fig. 1E,F and Additional file 1: Fig S3E,F). As a further step the distance of individual voxels from the nearest vessel within the tissue’s FOV were measured, transferred into a transformation map and evaluated (Fig. 1G,H and Additional file 1: Fig S3G,H). Segmentation, tracing and distance transformation of the organs’ vasculature, yielded complex data sets, containing a multitude of parameters describing not only the vessel network in its entirety but also each individual vessel segment and their spatial connection to the supplied tissue (Fig. 2A, Additional file 1: Table S2).

To examine similarities and differences in the vascular architecture of the normal organs these obtained complex data sets were subjected to principal component analysis (PCA, Fig. 2B). PCA revealed that the parameters of the vascular architecture varied reproducibly, and strongly enough between different tissue to allow for clear assignment to the various organs. This emphasized the fact that different organs need to address their individual metabolic needs and physiological functions by forming distinguishable vascular structures. Vessel density, and therefore relative perfused vessel volume (rPVV) varied strongly (between 3.9 ± 0.3% in muscle and 9.4 ± 0.4% in the colon’s mucosa) (Fig. 2C). Differences were found also in average length of vessel segments between bifurcations (between 68.5 ± 2.7 µm (muscle) and 37.7 ± 1.1 µm (colon)) and to a lesser degree in vessel diameter (between 8.2 ± 0.2 µm (muscle) and 13.2 ± 0.5 µm (colon)) (Fig. 2D, E). We observed clear differences in the orientation of the individual vessel segments. In muscle tissue we see long, linear and parallel vessels that largely orientate longitudinally to muscle fibers. Detailed analysis of vessel orientation angels revealed a most uniform direction in the muscle (Fig. 2D, Additional file 1: Fig S5A,B)). In contrast, the brain’s vasculature shows the opposite with short, arched vessels of arbitrary orientation. In the colon and retina, a tendency to preferred vessel orientation was less pronounced than in muscle, but still noticable. In the retina vessels form two connected two-dimensional meshes. The vasculature in the colon’s mucosa forms a single similar mesh directly beneath the epithelial layer lining the lumen (colon surface). The high average vessel diameters of vessels in this mesh (Additional file 1: Fig. S7B) are responsible for significant higher average vessel diameters in the colon overall, while the other healthy organs were rather similar in that regard. The mesh in the colon surface is connected to a dense pattern of parallel vessels longitudinal to the crypts.

### Vascular parameters in tumors

**Fig. 3:**
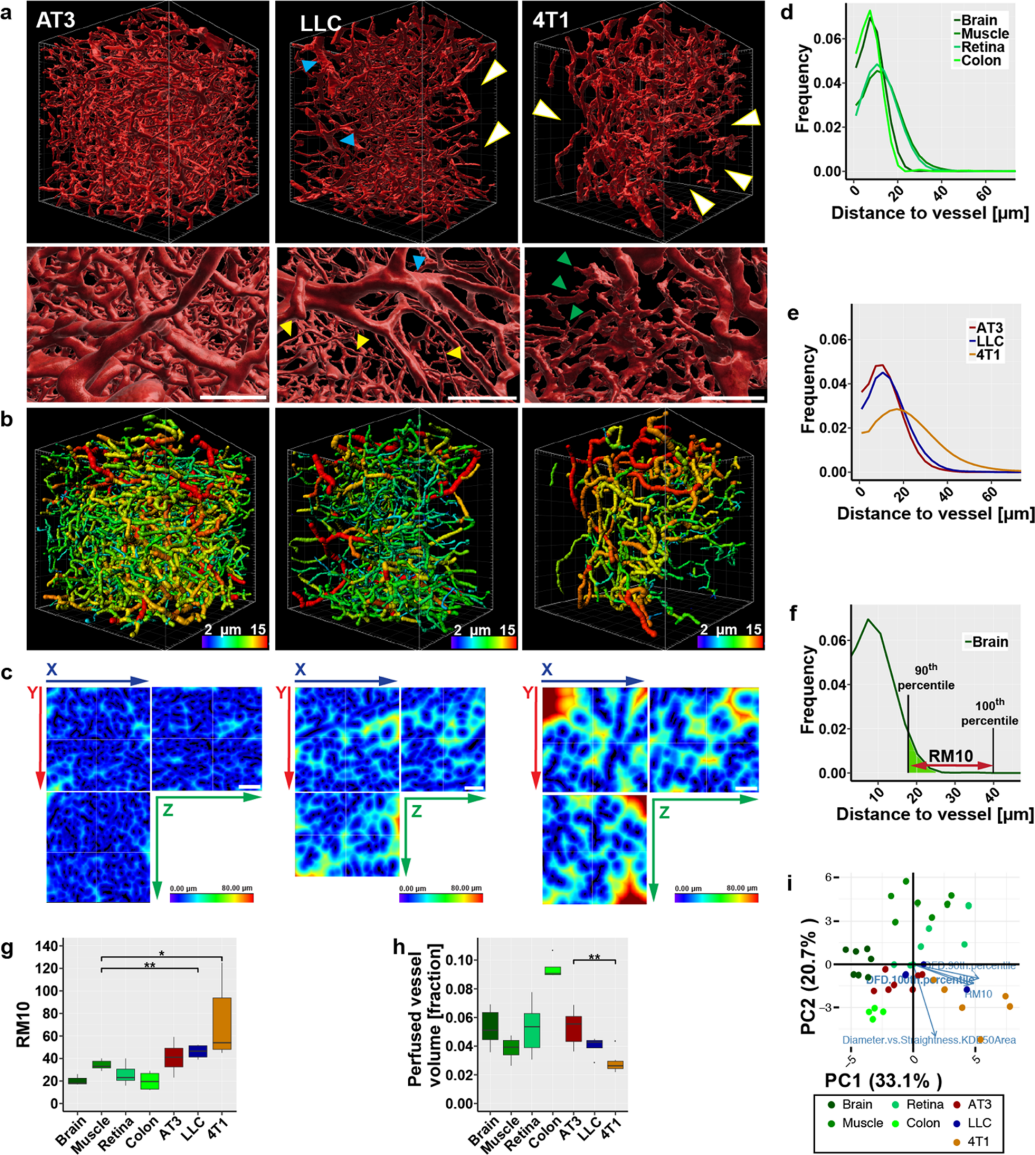
Comparison of selected intravital stained tumors with healthy organs. **a** Segmentation of the perfused vasculature in three different tumors. Detail showing the differences in surface structure of the vessels. White arrowheads: areas void of perfused vessels. Blue arrowheads: enlarged vessels. Yellow arrowheads: small capillaries. Green arrowheads: irregular protrusion on vessels in 4T1 tumors. Grid scale: 50 µm, SB: 100 µm. **b** Tracing results of perfused vessels in the respective tumor models. Vessel segments are displayed color-coded according to average diameters. Grid scale: 50 µm. **c** Heatmaps displaying distances from nearest perfused vessel in the respective tumors. SB: 100 µm. **d** Distance frequency distribution to closest perfused vessel in healthy organs. n ≥ 4. **e** Distance frequency distribution to closest perfused vessel in tumors. n ≥ 4. **f** Graphical explanation of the RM10 value on the example of the distance frequency distribution in the brain. **g** Comparison of RM10 values of normal organs and tumors. n ≥ 4. **h** Comparison of perfused vessel volume as fraction from total tissue volume of normal organs and tumors. n ≥ 4. **i** Results from PCA of vascular parameters in normal organs and in the three tumor models. Orientation of four highly ranked selected variables (blue arrows) are shown. Error bars: ± SEM. Asterisks display results from statistical tests with *: P < 0.05, **: P < 0.01, ***: P < 0.001, ****: P < 0.0001

To contrast the results from normal tissue three syngeneic murine tumors were selected: AT3 and 4T1 breast carcinomas and lewis lung carcinomas (LLC). Within the tumors, strong heterogeneity of vessel density was evident. The 3D-angiography of the vasculature in all three models revealed distinct aberrations from the blood vessels observed in normal organs (Fig. 3A). The uneven rendering of the vascular surface—most pronounced in the 4T1 model—was indicative of leakiness (Fig. 3A, A’). Moreover, within the fields of view, distribution of the perfused vessels was still strikingly heterogeneous: while in some parts the vessel density appeared extremely high, other parts were completely void of perfused vessel. This heterogeneity was pronounced strongest in 4T1 again, while AT3 showed a more regular, homogeneous vessel distribution. In all tumor models individual, often isolated vessels segments appeared dilated while other—still perfused—segments are narrow and seem constricted (Fig. 3A’, B). Capillaries in normal tissue were of a much more uniform diameter. Importantly the enlarger tumor vessels are often not bigger supplying vessels, as the dilated parts are not longer, continues sections but short segments only connected to parts of the network with much smaller diameters. We assessed the supply situation quantitatively by determining the distances of each voxel within the 3D FOV from the nearest blood vessel (Fig. 3C–E). In normal tissues the maximum measured distance from the nearest vessel was 66 µm (means 32–58 µm, depending on the organ). In contrast, within tumor tissue distances of up to 177 µm (mean 62–114 µm) were observed. The frequency distribution of the voxel-to-vessel distances contains quantitative information about the homogeneity of the spatial vessel distribution within the tissue: as a measure for this homogeneity the distance between the 90th and 100th percentile, in other words the range containing the maximal 10% of observations (RM10) can be used (Fig. 3F, G). Muscle showed the highest RM10 values in the group of normal organs. While AT3 showed tendencies to higher values, LLC and 4T1 showed significant higher RM10 values, indicative of a less homogeneous distribution of the vessels. Moreover, the values for the different tumor models varied, displaying the diversity of dysfunctional vessel phenotypes reflected in these murine models. The reduced homogeneity in vessel distribution in the tumors, was paralleled by a decreased overall rPVV (Fig. 3H).

### Effect of anti-angiogenic treatment on vascular parameters in tumors/normalization

The effect of antiangiogenic therapy on the established parameters of the microscopic tumor vasculature was tested with two agents: the receptor-tyrosine kinase inhibitor axitinib and the VEGF-A sequestering antibody mG6-31 [30]. We verified the effectiveness of the applied anti-angiogenic therapies, and importantly that samples were obtained in a phase were signs of normalization could be expected—i.e. during the previously described normalization window [17, 31]. Immunohistological and functional assays that are commonly used to detect changes associated with vascular normalization were used for validation. Immunological staining for CD31 on tumor sections confirmed an overall reduced vascular density after treatment (Additional file 1: Fig. S6A-C). The vasculature of both LLC and AT3 tumors showed increased coverage with NG2^+^-pericytes after mG6-31 treatment (Additional file 1: Fig. S6D–F). Interestingly, in contrast to mG6-31, axitinib reduced pericyte coverage in both models. Axitinib inhibits not only the RTKs of the VEGFR-family but also has a strong affinity to PDGFRs (IC_50_: 5 nM for PDGFR-α and 1.6 nM for PDGFR-β)[32]. PDGF/PDGFR signaling is important for pericyte maturation and inclusion into the vascular wall, explaining why under treatment with a potent PDGFR-inhibitor pericyte-EC contacts might be even further reduced [33, 34]. Reduced extravasation of serum bound Evans Blue demonstrated improved vessel patency (Additional file 1: Fig. S6G). Overall treatment efficacy was reflected in increased density of cleaved caspase 3^+^-cells (Additional file 1: Fig. S6H,I).

**Fig. 4:**
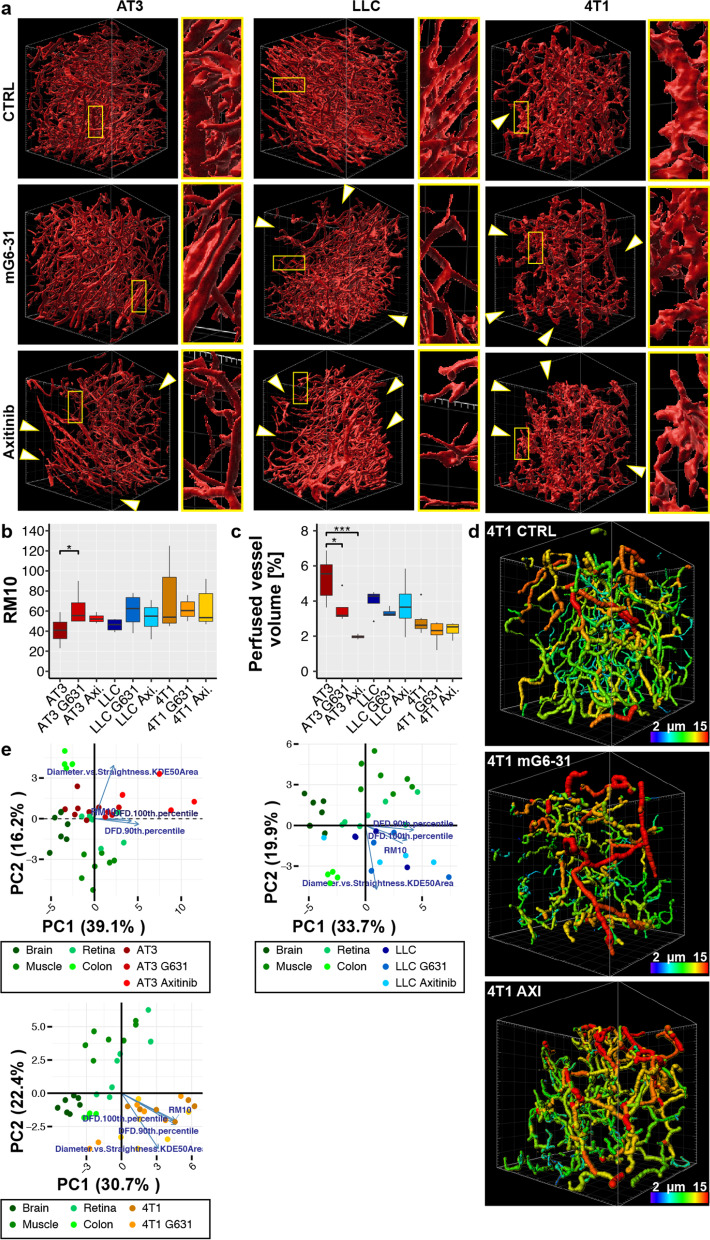
Effect of anti-angiogenic treatment on vasculature of tumors. **a** Segmentation of the perfused vasculature in different tumors including lineup of treatment results with the two anti-angiogenic agents mG6-31 and axitinib. White arrows indicate larger areas void of perfused vessels. Grid scale: 50 µm. **b** Comparison of RM10 values between tumors and tumors treated with mG6-31 or axitinib. n ≥ 4. **c** Comparison of relative perfused vessel volume between tumors and tumors treated with mG6-31 or axitinib. n ≥ 4. **d** Tracing results of perfused vessels in 4T1 tumors after treatment with mG6-31 or axitinib. Vessel segments are displayed color-coded according to average diameters. Treatment did not result in a more homogeneous distribution of vessel diameters, but overall vessel diameter was increased. Grid scale: 50 µm. **e** Results from PCA of vascular parameters in normal organs and in the three tumor models after anti- angiogenic therapy with either mG6-31 or axitinib. Orientation of four highly ranked selected variables (blue arrows) are shown. Error bars: ± SEM. Asterisks display results from statistical tests with *: P < 0.05, **: P < 0.01, ***: P < 0.001, ****: P < 0.0001

After verification that the treated samples showed all the signs commonly associated with vessel normalization, the vascular architecture was examined by 3D-angiography. The obtained images showed no evident change towards a more organized network after treatment in any of the tumor models (Fig. 4A). On the contrary, size and number of sections within the FOV void of blood vessels increased as a result of the anti-angiogenic pruning, while the vessel segments retained their random arrangements and interconnectivity. In LLC and 4T1 remaining vessels appeared to be clustering in confined areas. Accordingly, RM10 values, describing the heterogeneity of the vessel distribution, increased under treatment: significantly in AT3 tumors, moderately in LLC and 4T1 tumors (Fig. 4B). As expected the rPVV decreased (Fig. 4C), also not as strongly as indicated by the reduced vessel density observed in 2D sections (Additional file 1: Fig. S6A–C). This was at least in part due to an increase in enlarged vessels after treatment (Fig. 4D, Additional file 1: Fig. 7A–C). As the occupied volume of some vessel segments strongly increased it compensated in part for the reduced number of vessels. In line with the previous analyses, PCA also indicated that antiangiogenic treatment with either mG6-31 or axitinib did not move vascular parameters closer to those of normal tissue (Fig. 4E). On the contrary, in the tumor model that demonstrated the vascular architecture closest resembling those in normal organs, AT3, treatment with either of the drugs shifted parameters further from those in these normal organs.

**Fig. 5:**
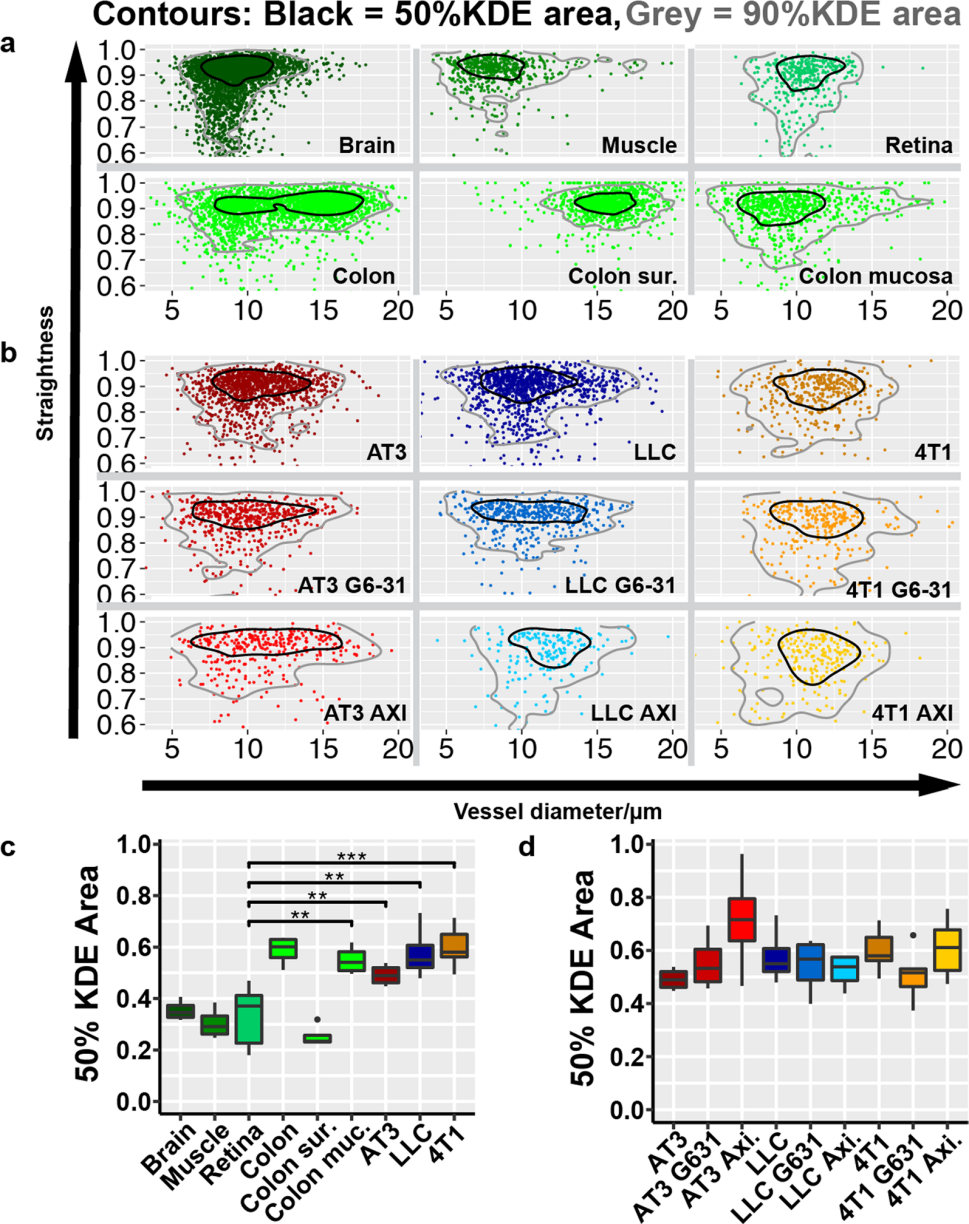
Analysis of vessel average diameter versus vessel straightness.** a** Scatterplot of vessel average diameters vs vessel straightness of healthy tissue.** b** Scatterplot of vessel average diameters vs vessel straightness of control tumors and after tumors after antiangiogenic treatment. Each panel shows only one representative tissue sample for clarity. Black line contours 50% kernel density estimate area (KDE area) and grey line contours 90% KDE area.** c** Comparison of the 50% KDE Area in healthy tissue and in tumor tissue. n ≥ 4.** d** Comparison of the 50% KDE Area in tumor tissue and in tumor tissue after anti-angiogenic treatment with mG631 or Axitinib. n = 4–6. Error bars: ± SEM. Asterisks display results from statistical tests with *: P < 0.05, **: P < 0.01, ***: P < 0.001, ****: P < 0.0001

To add information about the correlation between vessel length, straightness and diameter, we compiled 2-dimensional kernel density estimates (KDE) for these characteristics pairwise and calculated the 50% KDE area (Fig. 5A–C, Additional file 1: Fig. S7A). Diameter vs. straightness showed significant smaller KDE areas (KDE_50(D/S)_) in normal organs compared to tumor tissues with the only exception being the colon. In separate inspection, the surface vessel mesh of the colon also showed significant lower KDE_50(D/S)_ areas while the remaining vessels did not (Additional file 1: Fig. S7B). Importantly, treatment with axitinib or mG6-31 did not have a significant effect on the size of KDE_50(D/S)_ areas in any of the observed tumors (Fig. 5D). KDE areas of length vs any other characteristic showed results with no obvious distinctions between healthy tissues and tumors (Additional file 1: Fig. S7C).

Comparing the effect of the angiogenic treatment in the three models, it is apparent that the strongest effect on the vessel architecture can be observed in AT3 tumors, the model originally displaying the densest and most organized vasculature, most similar to those in normal organs. However, the effect of the anti-angiogenic treatment was clearly detrimental, towards a more unorganized architecture (Fig. 5D). In LLC and 4T1 tumors, with their already problematic vasculature, anti-angiogenic treatment also had not an improving effect, essentially leaving the tissue with less of the same unorganized vasculature. Thus, anti-angiogenic treatment is driving the initially distinct architecture of the tumors towards a uniformly inhomogeneous and highly dysfunctional status.

## Discussion

Tumors display characteristics that directly interfere with the efficacy of therapeutic approaches. A sensible approach is to strategically remove these obstacles in order to increase therapeutic efficacy and reduce side effects. The defectiveness of the vasculature in many solid tumors has long been understood as an impediment for drug delivery and an ultimate cause for increased malignancy, invasiveness and metastasis as a response to ill supply. Improving or repairing the tumor vasculature and the application of anti-angiogenic drugs is discussed for years as a suitable method.

To enable a coherent approach to vascular improvement for increased supply, it is necessary to understand first the parameters that determine the capacity of a vascular system, and next how to influence them interdependently. The distinct structures of the vasculature from different organs have emerged out of long evolutionary processes and are constructed in tightly regulated developmental processes. The vasculature in the broad spectrum of normal organs represents the logical standard against which the defectiveness of the tumor vasculature and any pharmacologically induced corrective effects should be measured. Unbiased approaches to determine unifying determinants in the vascular architecture of various organs, led us focusing on two aspects, the hierarchical structure of the networks and their homogeneity. We found the hierarchies of the vascular networks in normal tissue to be reflected in the KDE of vessel diameter vs. straightness. Small KDE_50(D/S)_ areas are the result of only few large vessels splitting into smaller vessels of large numbers successively leading overall to less variability in diameters. Similarly, the margin for the straightness of vessels is reasonably small in normal tissue, since they rarely form steep curves or even loops.

In tumors the regulation of angiogenesis is disturbed and therefore vessel sprouting, formation and maturation occurs in a poorly regulated, chaotic manner. Diameter, straightness or both range over a wider margin. AT3 tend to higher variability in the diameters and anti-angiogenic treatment increased the variability even more. In LLC mG6-31 treatment clearly affected the shape of the KDE_50(D/S)_ by increasing the variability in vessel diameters even more but also by decreasing the variability in vessel straightness. Axitinib in contrast had the exact opposite effects. Together those effects lead to no overall change in the KDE_50(D/S)_ area in LLC. In fact, both treatments failed to improve the KDE_50(D/S)_ areas of any of the regarded tumor models, despite some changes in either diameter, straightness or both. Conclusively low KDE_50(D/S)_ values are indicators for better hierarchies in the vasculature, a premise for efficiency.

Another important factor for efficiency in the vascular network is the homogeneity of the blood vessel distribution. The extra-vascular transport of oxygen and nutrients relies on diffusion through cells or interstitial fluids [35]. The diffusion limit for oxygen depends strongly on the characteristics of the extra cellular space, but a theoretical values range between 100 and 200 µm [36, 37]. Within this limit cells have to located to survive. In an efficient vascular network blood vessel distance from cells should be minimized, while the total volume for the network has to be reasonable small. This results in a homogenous distribution of vessels and guarantees that even in tissue with low rPVV all areas are in sufficient close proximity of a supplying vessel. Our data showed that in normal organs the distance to the next blood vessels is always under 70 µm while in tumor almost up to three times that distance can be observed. Cells located at these distant spots are likely supplied insufficient, an observation in accordance with the established data that in most solid tumors strongly hypoxic and under-supplied regions exist [38, 39]. Parameters describing the homogeneity of a vascular network are therefore essential, to judge the ability of the network to efficiently supply the surrounding tissue. Distance of maximally remote spots, is indicative of problems with supply, but unsuitable to compare vessel homogeneity in various tissues, as it is dependent on rPVV. We found the RM10 value to be a good measure for describing vascular homogeneity. Low RM10 values indicate homogeneous distributed blood vessels. AT3-tumors had RM10 values close to normal tissue. Under anti-angiogenic therapy, the RM10 values increased. In 4T1-tumors and LLC anti-angiogenic therapy did not alter the rPVV or the RM10 values significantly. That demonstrates how a given anti-angiogenic therapy does not affect every tumor vasculature in the same way. In fact, our results seem to indicate that in tumors with a fairly adequate vascular architecture anti-angiogenic therapy indeed reduces this adequacy, resulting in increased under-supply. In tumors with an already dysfunctional vasculature (4T1, LLC) anti-angiogenic therapy seem not to further harm supply to this extend. But importantly, all our data contradict the concept that anti-angiogenic therapy “normalizes”—i.e. brings the vascular parameters closer to those in normal tissue—the tumor vascular architecture. However, it might explain why anti-angiogenic therapy shows strongly variable efficacy: in tumors with high rPVV, anti-angiogenics can indeed fulfill their initially intended task of starving the tumor, while this effect is strongly reduced in tumors already undersupplied by a malformed vasculature. Indeed, best results with stand-alone antiangiogenic therapy were reported from highly vascularized cancers like RCC [40, 41].

The possibility to rank the effect of a treatment—not necessarily antiangiogenic, as other drugs have also shown profound, often indirect effects on the vasculature—by two simple parameters KDE_50(D/S)_ and RM10 allows for a methodical improvement of treatment modalities. Maybe vascular normalization can thereby become a mainstay in a more strategic approach to cancer therapy.

## Materials and methods

### Reagents

Chemicals were acquired from standard commercial suppliers (SigmaAldrich, Merck).

All experiments involving animals were reviewed and approved by the Regierung von Unterfranken, Würzburg (Germany). The experiments were performed in accordance with relevant guidelines and regulations.

### Cell culture

LLC and 4T1 cells were obtained from ATCC. AT3 cells were obtained from SigmaAldrich. All cell lines were maintained in DMEM with 10% FBS and penicillin/streptomycin. Cell lines were tested at least every three months for mycoplasma contamination.

### Clearing media and procedures

Animals were injected 20 min prior to sacrifice with 10 µg Alexa-647 labeled CD105 antibody and killed by CO_2_ asphyxiation [42].

#### Fixation

Euthanized mice were perfused through the heart with 10 ml PBS, followed by 10 mL of 4% (w/v) PFA in PBS. Organs and tumors were removed and submersion-fixed for 24 h at 4 °C in 4% (w/v) PFA in PBS. Excess PFA was removed with two changes of PBS for 24 h at 4 °C.

#### Decolorization

*CUBIC1 reagent *[25]: 25% urea, 25% (w/v) N,N,N′,N′-tetrakis(2-hydroxypropyl)ethylenediamine (Quadrol), and 15% Triton X-100 in water.

For decolorization, organs and tumors were immersed in a 10–20-fold excess of CUBIC1 at 37 °C with shaking for 72 h. The reagent was exchanged for an equal volume of CUBIC1 and incubation was continued for another 72 h. Organs were washed in several changes of PBS for 72 h before further processing.

The procedure for decolorization with Quadrol was performed analogous, only 25% (w/v) N,N,N′,N′-tetrakis(2-hydroxypropyl)ethylenediamine (Quadrol) in water was substituted for the CUBIC1 reagent.

*Hydrogen peroxide decolorization reagent *[26]: 20% (v/v) H_2_O_2_ (30%), 20% (v/v) DMSO, 60% (v/v) methanol.

For decolorization, organs and tumors were first subjected to a gradual dehydration in methanol (50%, 80% and 100% MeOH for at least 4 h at r.t.) and then placed in a 10- to 20-fold excess of the H_2_O_2_-decolorization reagent at 4 °C with shaking for 24 h. Samples were than washed twice with methanol for 4 h at r.t. and either stored in methanol or rehydrated (80% MeOH (4 h), 50% MeOH (4 h) and PBS ON) and processed.

#### Clearing

*CUBIC2 reagent *[25]*:* 50% (w/v) sucrose, 25% (w/v) urea, 10% (w/v) 2,2′,2′-nitrilotriethanol, and 0.1% Triton X-100 in water. Sucrose and urea were dissolved in water by stearing at 37 °C. 2,2′,2′′-nitrilotriethanol and Triton X-100 were added. The reagent was stored at 37 °C.

For clearing the organs were first immersed in 20% (w/v) sucrose in PBS for 24 h at 4 °C, and then placed in a 10–20-fold excess of CUBIC2 at 37 °C with shaking for 72 h. The reagent was exchanged for an equal volume of CUBIC2 and incubation was continued for at least another 72 h or until the organs appeared transparent.iDISCO [26]: For clearing the organs were first dehydrated in THF (50% THF in water (4 h, r.t.), 80% THF in water (4 h, r.t.), 100% THF (ON, r.t.)) and then placed in excess of dichloromethane (DCM) for at least 24 h or until the tissue sank. DCM was then replaced by dibenzylether (DBE) and incubated at r.t. for 24 h after which DBE was exchanged. Tissue sections were kept in DBE until imaging.

Ethyl cinnamate [27]: For clearing the organs were first dehydrated in EtOH (50% EtOH in 10 mM Tris HCl pH 9.0 (4 h, 4 °C), 70% EtOH in 10 mM Tris HCl pH 9.0 (4 h, 4 °C), 90% EtOH in 10 mM Tris HCl pH 9.0 (4 h, 4 °C), 96% EtOH (4 h, 4 °C), 96% EtOH (4 h, 4 °C), 100% EtOH (100 h, 4 °C), 100% EtOH (4 h, 4 °C),) and then placed in excess of ethyl cinnamate (EtCi) for at least 24 h at r.t. after which EtCi was exchanged. Tissue sections were kept in EtCi until imaging.

### Tumor models and treatment

*Tumor engraftment:* LLC (1 × 10^6^ cells in matrigel/PBS 1:1) tumors were generated by subcutaneous injection in the dorsal region of female C57Bl/6 J mice. 4T1 (1 × 10^5^ cells in PBS) breast adenocarcinomas were generated by injection of cells into the inguinal mammary fat pad of female Balb/c mice. EMT6 (1 × 10^6^ cells in PBS) breast adenocarcinomas were generated by injection of cells into the inguinal mammary fat pad of female Balb/c mice. AT3 (1 × 10^6^ cells in PBS) breast adenocarcinomas were generated by injection of cells into the inguinal mammary fat pad of female C57Bl/6 J mice.

All animals in the individual experiments were of the same age and sex. For each experiment tumor bearing mice were randomly assigned to the different treatment groups just prior to the start of treatment.

### Miles assay for vascular permeability

Anesthetized mice were injected retroorbital with 50 µL of an 2% (w/v) solution of Evans Blue in 0.9 sterile saline. Animals were sacrificed 30 min later, perfused with PBS through the left ventricle, before organs were removed and stored at -80 °C until later processing. At least three samples from different parts of individual tumor were harvested to account for the strong heterogeneity in this tissue. Extravasated Evans Blue was extracted by adding 9 parts (w/v) of formamide to the tissue samples and incubation at 60 °C for 24 h. Absorption was measured at 620 nm and 740 nM. After correcting values for co-extracted hemoglobin (A_620nm_ (corrected) = A_620nm_—(1.426 × A_740nm_ + 0.030). Organ concentrations were calculated by comparison with a standard curve [43].

### IHC and IF staining of tumor sections

H&E, IHC and IF staining was performed using standard techniques on formalin fixed paraffin embedded sections. Tissues for quantitative evaluation were processed in parallel. For quantification whole tissue sections were imaged on a Keyence BD 6000 microscope with an automated stage using a Nikon 10 × objective. The individual images were stitched using the Keyence Analyzer software, to obtain a virtual slide. The whole virtual slide was used for quantification using the ImageJ software package (*rsbweb.nih.gov/ij/*).

Immunofluorescence images were acquired on a Nikon A1 laser-scanning confocal microscope using a Nikon 20 × objective. Image processing and quantification was performed using the ImageJ software package (*rsbweb.nih.gov/ij/*).

Antibodies used for IHC, IF or WB: Cleaved Caspase-3 (Cell Signaling Technology Cat# 9661, RRID:AB_2341188), Carbonic Anhydrase IX (Santa Cruz Biotechnology Cat# sc-25599, RRID:AB_2066539)), CD31 (Santa Cruz Biotechnology Cat# sc-28188, RRID:AB_2267979), CD34 (Abcam Cat# ab8158, RRID:AB_306316), Ki67 (Abcam Cat# ab16667 RRID:AB_302459).

### LSFM image acquisition

#### LSFM setup

A custom-built LSFM setup tailor-made for organ imaging was used: a customized fiber-coupled laser combiner (BFI OPTiLAS GmbH, Groebenzell, Germany) provided the required excitation lines of 491, 532, 642, and 730 nm. For laser beam collimation two objectives (RMS10X-PF Thorlabs, Bergkirchen, Germany) for VIS and 730 nm, were used. A DCLP 660 dichroic beam splitter (AHF Analysentechnik, Tübingen, Germany) combined the two beam paths. A following telescope (BEX 1x-4 × 017052-202-26, Jenoptik, Jena, Germany) served to adjust beam diameter. Alternating dual-side illumination was realized by a two-axis galvanometer scanner (6210H; Cambridge Technologies, Bedford, MA, USA) in combination with a theta lens (VISIR f. TCS-MR II; Leica, Mannheim, Germany) which finally created a virtual light sheet that was additionally pivot scanned by a single-axis resonant scanner system (EOP-SC, 20-20 × 20-30-120; Laser2000, Wessling, Germany) to minimize shadowing artifacts. The light sheet was projected onto the sample via a 200 mm tube lens (TTL200,Thorlabs, Bergkirchen, Germany) and a lens objective (Nikon CFI60 TU Plan Epi 5 × /0.15, Edmund Optics, York, United Kingdom). The objective on the detection side (HCX APO L 20 × /0.95 IMM; Leica, Mannheim, Germany) placed on a piezo positioning system (P-611.1 and E-665, PI, Karlsruhe, Germany) for focus correction collected the fluorescence perpendicularly to the light sheet, and, in combination with an infinity-corrected 1.3 × tube lens (model 098.9001.000; Leica, Mannheim, Germany), projected the image into a scientific complementary metal oxide semiconductor (sCMOS) camera (Neo 5.5; Andor, Belfast, United Kingdom) (2,560 by 2,160 pixels, 16.6-mm-by-14.0-mm sensor size, 6.5-μm pixel size). The fluorescence was spectrally filtered by typical emission filters (AHF Analysentechnik, Tübingen, Germany) according to the use of the following fluorophores: BrightLine HC 525/50 (Alexa Fluor 488 or autofluorescence), BrightLine HC 580/60 (Alexa Fluor 532), HQ697/58 (Alexa Fluor 647), BrightLine HC 785/62 (Alexa Fluor 750). Filters were part of a motorized filter wheel (MAC 6000 Filter Wheel Emission TV 60 C 1.0 × with MAC 6000 controller; Zeiss, Göttingen, Germany) placed in the collimated light path between detection objective and tube lens.

### Image acquisition

Cleared organ and samples were placed in the light path within the EtCi-filled objective chamber using a clamp-holder. Stacks were acquired in increments of 1 μm by imaging each plane in two color channels (BrightLine HC 525/50 (autofluorescence), and HQ697/58 (Alexa Fluor 647)) sequentially. Typically stacks of 1,024 × 1,024 × 400–700 voxels with a voxel size of 0.5 × 0.5 × 1 µm^3^ were acquired. Hardware components for image acquisition (laser, camera, filter wheel, stage, focus correction) were controlled by IQ 2.9 software (Andor, Belfast United Kingdom). Images were saved as tagged image files (TIF), processed and analyzed as described below.

### Image analysis

#### Image pre-processing

Acquired raw tiff-image stacks were loaded into the Fiji-distribution of ImageJ (https://fiji.sc/). As stack parameters, were routinely not correctly read-in from image meta data, they were manually corrected. The stack was processed using Fiji’s 3D-median filter by replacing each pixel value with the median of two neighboring pixel values. The processed stack was saved as singled tif file and converted using the Imaris file converter in an Imaris-9.8-file. Converted files were read into Imaris (Version 9.8.0, Oxford Instruments, Abington, UK).

#### 3D-reconstruction

The vascular network was reconstructed in the software package Imaris. A surface was created with standardized settings (Additional file 1: Table S2), although threshold levels for the used fluorescent signal had to be individually adjusted to compensate for varying staining and background intensities in the different organs. The generated surface was cleaned up by filtering fragments smaller than 2000 vx. Tabular results of the calculated surface parameters were exported in form of spreadsheets.

#### Tracing

The pre-processed fluorescence signal proofed in some organs too clouded with background for successful tracing of the vasculature. Therefore, the fluorescence signal was cleaned by masking using the previously reconstructed surface: the fluorescence signal outside the vessel surface was set to zero to remove background and small artifacts. Using the *filament* creation tool, the thereby generated new masked channel was utilized to trace the blood vessel signal. Standardized settings were used for generating traces (Additional file 1: Table S3). The imaging procedure by restricting the analyzable volume to a small FOV of high resolution, resulted necessarily in vessels cut-off at the rim of the FOV. These partial vessel segments would falsify the data and were therefore removed before statistical analysis. Tabular results of the calculated vessel parameters were exported in form of spreadsheets.

(Side note: the “*filament tool*” was developed for analysis of dendritic trees. Accordingly, the results are described with unusual terms: “Dendrites” for individual segments between two bifurcations/branching points; “Filaments” for connected networks of individual segments (“Dendrites”)).

#### Distance transformation

The *Matlab-*based (MathWorks, Natick, MA) distance transformation routine in Imaris was used to first visualize distance of individual voxels to the surface of nearest vessel structure. This generated a new 8-bit channel in the 3D-stack in which distance to the vessel surface was decoded by a grey value in the range of 0–255. The channel was exported to Fiji. Using the histogram of the 3D-stack allowed export of the visual data into numerical values for further analysis. For visualization the grey values of the channel were spectral color-coded.

### Statistical analysis

Statistical analysis and PCA was done using the R environment (https://www.r-project.org/). Differences between two groups were analyzed using an unpaired, two-tailed Student’s T-test. In parallel the samples were tested for significant variation of variance, and if necessary a Welch correction was included in the statistical analysis. All statistical tests were performed between sets of individual biological replicates.

## Supplementary Information

**Additional file 1.** Supplemental Data.

## Data Availability

Not applicable.
