# Supplementary material for: Image-based modeling of vascular organization to evaluate anti-angiogenic therapy
Source: Biol Direct. 2023 Mar 15;18:10. doi: 10.1186/s13062-023-00365-x (PMC10018970; doi:10.1186/s13062-023-00365-x)
Supplement: Supplementary file 1 — Additional file 1. Supplemental Data. [file 13062_2023_365_MOESM1_ESM.docx]

**Image-based modeling of vascular organization to evaluate anti-angiogenic therapy**

David Ascheid^1^, Magdalena Baumann^1^, Caroline Funke^1^, Julia Volz^2,3^, Jürgen Pinnecker^3^, Mike Friedrich^3^, Marie Höhn^1^, Rajender Nandigama^1^, Süleyman Ergün^1^, Bernhard Nieswandt^3^, Katrin G. Heinze^3^*, Erik Henke^1,4^*

**- Supplemental Data -**

^1^ Institute of Anatomy and Cell Biology, Universität Würzburg, Würzburg, Germany.

^2^ Institute of Experimental Biomedicine I, Universitätsklinikum Würzburg, Würzburg, Germany.

^3^ Rudolf Virchow Center for Integrative and Translational Bioimaging, Universität Würzburg, Würzburg, Germany.

^4^ Graduate School for Life Sciences, Universität Würzburg, Würzburg, Germany.

* To whom correspondence should be addressed:

Katrin Heinze, Dr. rer. nat.

Chair of Molecular Microscopy

Rudolf Virchow Center for Integrative and Translational Bioimaging

Universität Würzburg

Josef-Schneider-Straße 2

97080 Würzburg, Germany.

[katrin.heinze@virchow.uni-wuerzburg.de](mailto:katrin.heinze@virchow.uni-wuerzburg.de)

Tel: +49-(0)931-3184214

Erik Henke, PhD

Institute of Anatomy and Cell Biology

Universität Würzburg

Koellikerstrasse 6

97070 Würzburg, Germany

Email: [erik.henke@uni-wuerzburg.de](mailto:erik.henke@uni-wuerzburg.de)

Tel: +49-(0)931-3183270

| **Supplemental table 1: Conditions for decolorization and optical clearing** | | | | | | | | |
| --- | --- | --- | --- | --- | --- | --- | --- | --- |
| **Fixation** | 4 % PFA in PBS, 4 °C, 24 h | | | | | | | |
|  | PBS, 4 °C, 24 h  Tumors: cut into blocks of ca. 5 x 5 x 3 mm | | | | | | | |
| **Pre-treat-ment** | 50% MeOH, r.t. 4h  80% MeOH, , r.t. 4h  100% MeOH,, r.t. 4h | - | | | | | | |
| **Decolorization** | H_2_O_2_/DMSO/MeOH  4°C  24 h | CUBIC1 reagent  37 °C,  2x 72 h shaking | | 20% CUBIC1  In 0.9% NaCl  37 °C,  2x 72 h shaking | 25% Quadrol  In 0.9% NaCl  37 °C, 2x 72 h shaking | | 5%  Quadrol  In 0.9% NaCl  37 °C, 2x 72 h shaking | PBS |
| **Storage** | 100% MeOH  4 °C | - | | | | | | |
| **Pre-treat-ment** | PBS, 24 h  3 changes | PBS, 72 h  3 changes | | | | | | - |
| **Immunofluorescence staining** | | | | | | | | |
| **Optical Clearing** | **CUBIC2** | | **iDISCO** | | | **Ethyl Cinnamate** | | |
|  | 20% sucrose, degassed  CUBIC2 reagent  37 °C,  2x 72 h shaking | | 50 % THF, r.t. 4 h  80 % THF, r.t. 4h  100 % THF, r.t. ON  DCM, r.t., 24 h  DBE, r.t. 24 h | | | 50% EtOH, pH 9.0, 4°C 4h  70% EtOH, pH 9.0, 4°C 4h  90% EtOH, pH 9.0, 4°C 4h  96% EtOH, 4°C 2h  2x 100% EtOH_abs_, 4°C, 24h  2x EtCi, r.t., 24 h | | |
| DCM: dichloro methan; DBE: dibenzyl ether, EtOH: ethanol; EtCi: ethyl cinnamate; MeOH: methanol; Quadrol: *N,N,N’,N’*-tetrakis-(2-hydroxypropyl)ethylenedi- amine; THF: tetrahydro furane. | | | | | | | | |

| **Supplemental Table 2: Exemplary parameters for surface reconstruction of blood vessel in Imaris** |
| --- |
| *General* |
| Enable Region of Interest = false |
| Enable Region Growing = false |
| Enable Tracking = false |
| Enable Shortest Distance = true |
| *Source Channel* |
| Source Channel Index = 1 |
| Enable Smooth = true |
| Surface Grain Size = 1.00 um |
| Enable Eliminate Background = true |
| Diameter of Largest Sphere = 7.50 um |
| *Thresholding* |
| Enable Automatic Threshold = false |
| Manual Threshold Value = 50 (individually adjusted) |
| Active Threshold = true |
| Enable Automatic Threshold B = true |
| Manual Threshold Value B = 1621.74 |
| Active Threshold B = false |
| *Filtering* |
| "Number of Voxels Img=1" above 2000 |

| **Supplemental Table 3: Exemplary parameters for tracing of blood vessel in Imaris** |
| --- |
| *General* |
| Name = Threshold (loops) |
| Track (over time) = false |
| *Preprocessing* |
| Channel Index = 3 |
| Enable Preprocessing = false |
| Approximate Diameter = 4.00 um |
| Preserve Edges = false |
| *Segmentation* |
| Fill Cavities = true |
| Connected BaseLine = true |
| Threshold Low = 50.000 |
| Threshold High = 300.000 |
| *Graph Compilation* |
| Branch Length Ratio = 5.000 |
| Find Dendrite Beginning Point = false |
| *Finish* |
| Build all Time Points = true |
| Delete Working Channel = true |

**
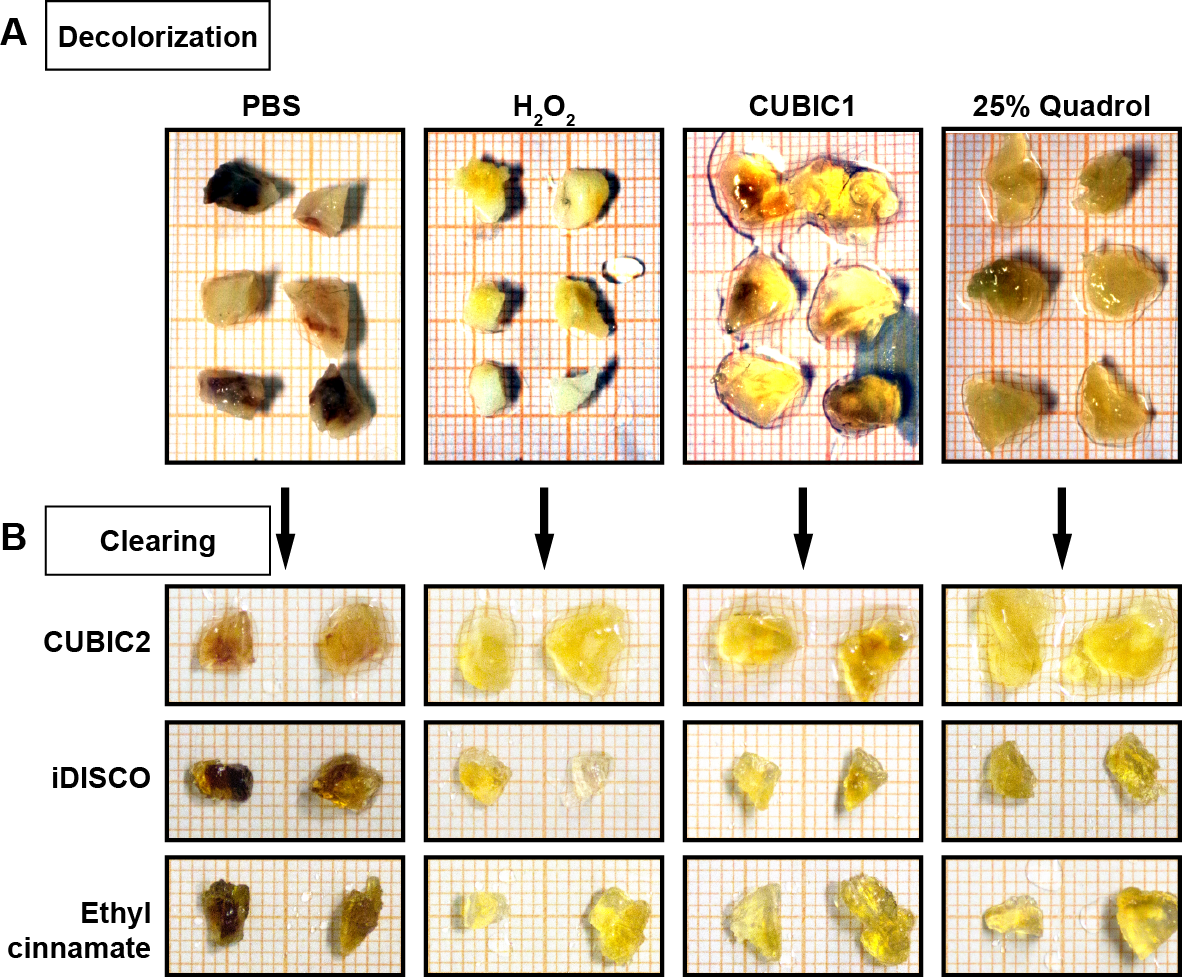
**

**Supplemental Figure S1: Comparison of decolorization and clearing procedures**

1. Segments of 4T1 murine carcinomas after application of different procedures for decolorization. For details of the procedures see Supplemental Table 1.
2. Segments of 4T1 murine carcinomas after application of different procedures for and clearing. For details of the procedures see Supplemental Table 1.

**
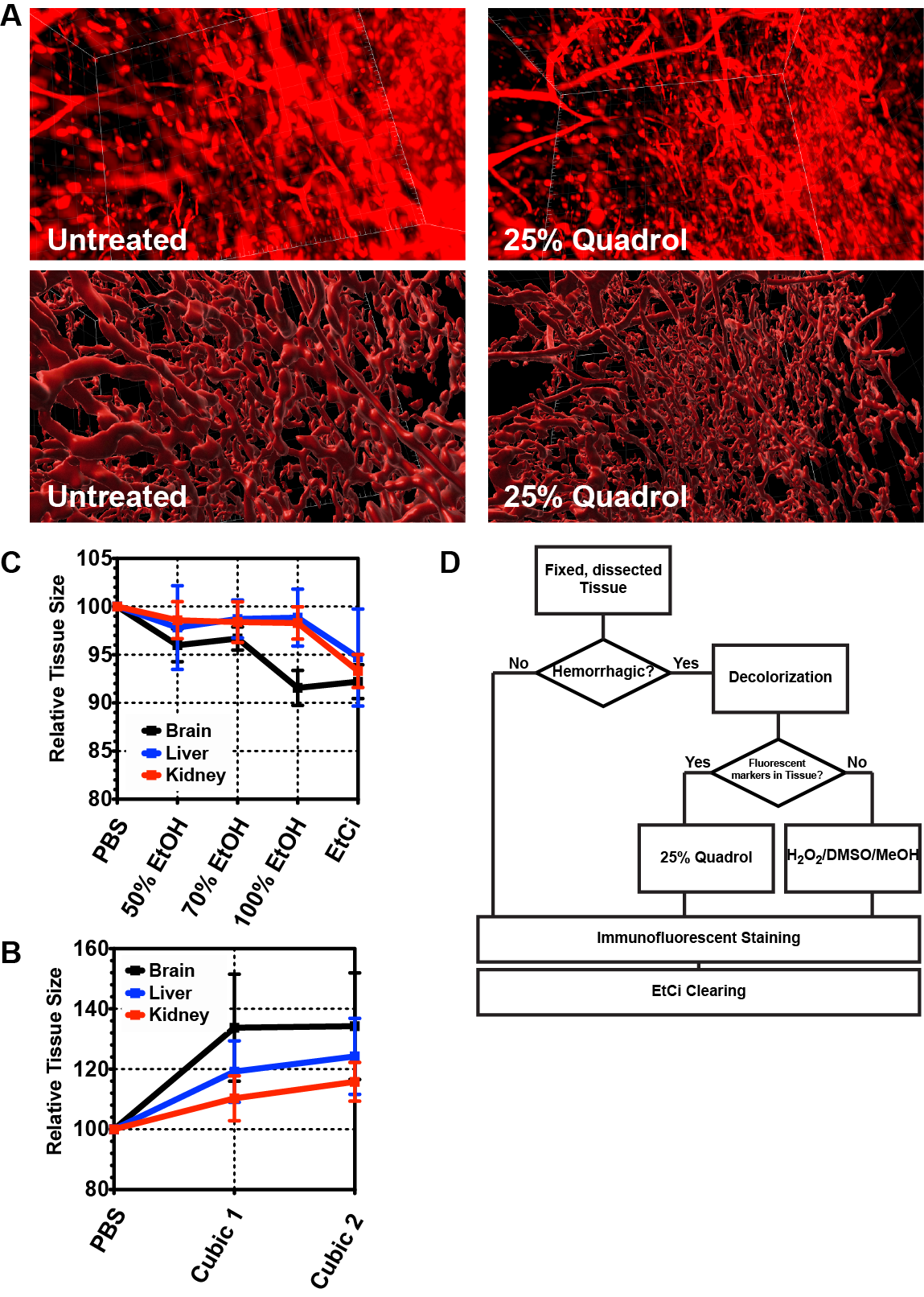
**

**Supplemental Figure S2: Tissue and fluorescent label preservation during decolorization and clearing**

1. Lungs decolorized by a 72h treatment with 25% Quadrol at 37 °C vs an untreated lung. Blood vessels were labeled by injection of Alexa 647-labeled CD105 antibody prior to dissection. The fluorescent label was retained during Quadrol treatment. Upper panels: unprocessed fluorescent signal, lower panels: segmented vessels. Grid scale: 50µm.
2. Volume changes during step-wise dehydration and clearing with ethyl cinnamate. Volume of the respective tissues was calculated from perpendicular measurements with a caliper in three directions.
3. Volume changes during step-wise clearing using the CUBIC procedure. Volume of the respective tissues was calculated from perpendicular measurements with an caliper in three directions.
4. Schematic workflow depending on needs for decolorization and fluorescence preservation.

**
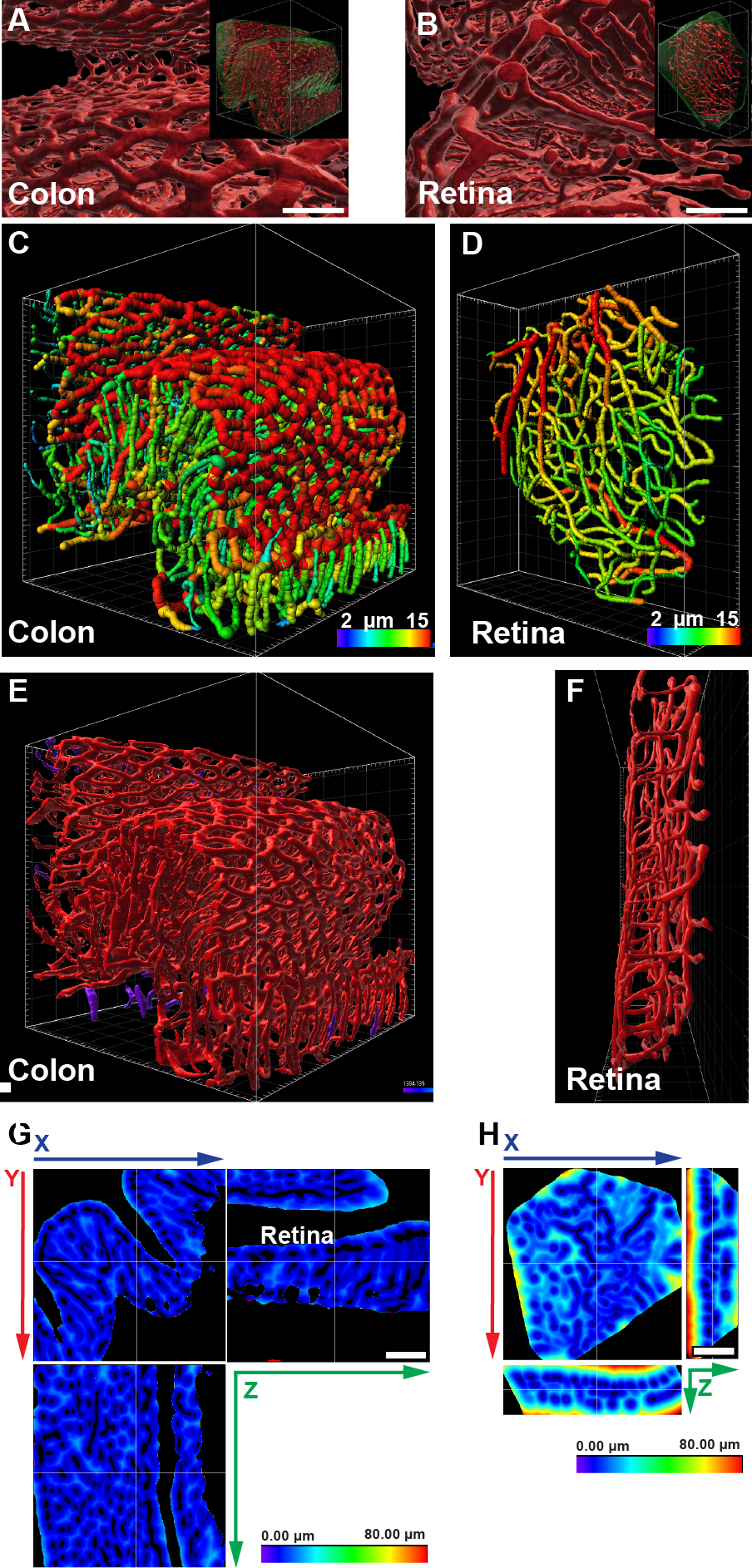
**

**Supplemental Figure S3: Vascular structures in normal organs.**

1. Representative 3D-projection of the perfused vasculature in the mucosa of the murine colon after segmentation. SB: 100µm. Insert: full representation of the tissue fragment acquired by LSFM. Grid scale: 50µm.
2. Representative 3D-projection of the perfused vasculature in the murine retina after segmentation. SB: 100µm. Insert: full representation of the tissue fragment acquired by LSFM. Grid scale: 50µm.
3. Tracing results of perfused vessels in the murine colon. Vessel segments are displayed color-coded according to average diameters. Grid scale: 50µm; Color scale: Average vessel segment diameter: 2-15µm
4. Tracing results of perfused vessels in the murine retina. Vessel segments are displayed color-coded according to average diameters. Grid scale: 50µm; Color scale: Average vessel segment diameter: 2-15µm
5. Rendering of segmented vasculature in the murine colon. The majority of vessels in the displayed cubic section forms an interconnected structure (red). On the surfaces of the displayed structure are few vessel segments (purple) observable that are not connected within the visible volume to the bulk of vessels. Grid scale: 50µm.
6. Rendering of segmented vasculature in the murine retina, viewed in parallel to the *stratum nervosum*. In this perspective the two parallel highly-interconnected vessel layers are immediately recognizable. Grid scale: 50µm.
7. Heatmap display of distances from nearest perfused vessel in the murine colon. SB: 100µm
8. Heatmap display of distances from nearest perfused vessel in the murine retina. SB: 100µm


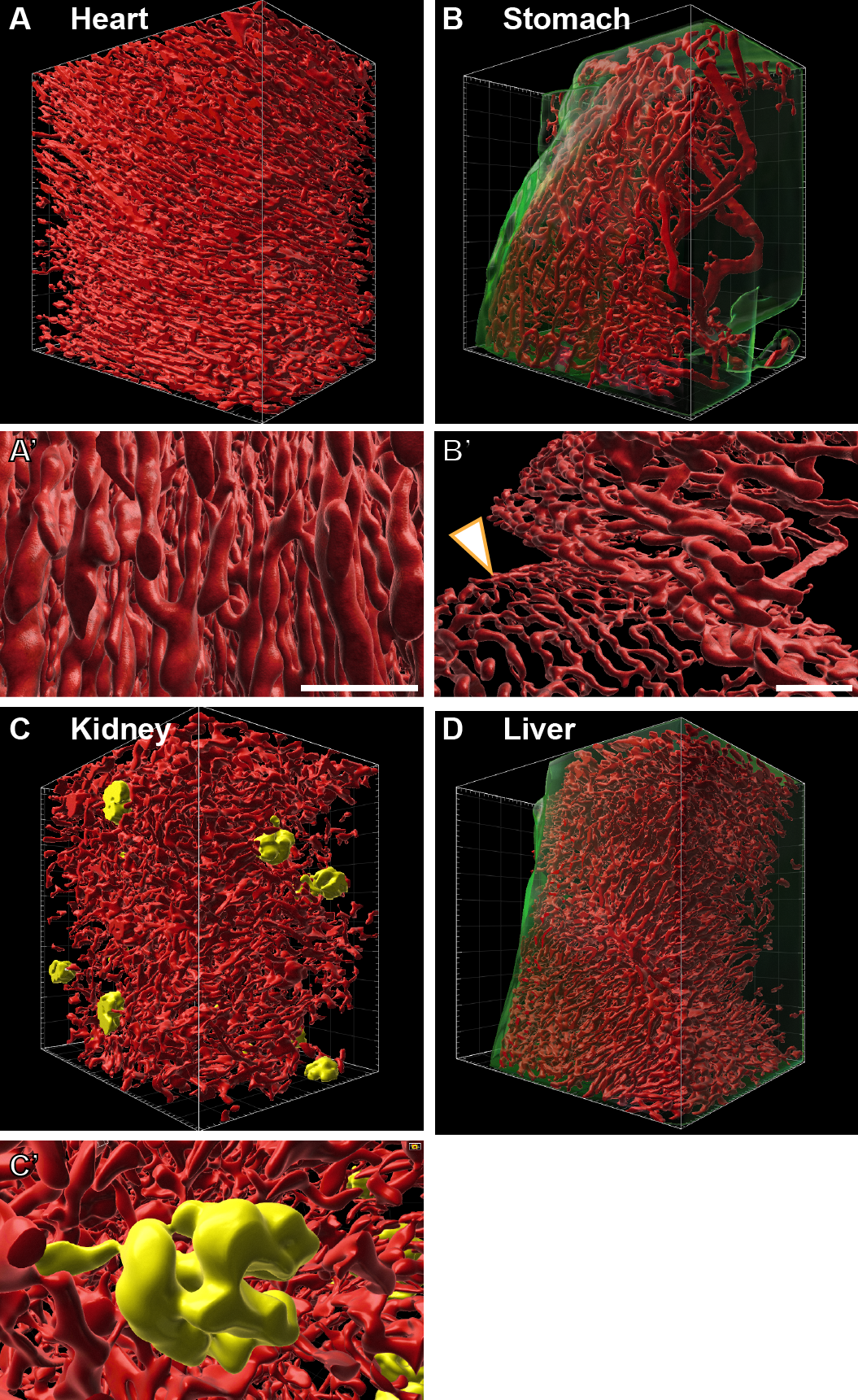


**Supplemental Figure S4: Vascular structures in additional normal organs.**

1. Representative 3D-projection of the perfused vasculature in the epicardium of the murine heart after segmentation. Grid scale: 50µm. (**A’**) Detail of the same 3D-rendering. SB: 100µm.
2. Representative 3D-projection of the perfused vasculature in the mucosa of the murine stomach after segmentation. Grid scale: 50µm. (**B’**) Detail of the same 3D-rendering. SB: 100µm.
3. Representative 3D-projection of the perfused vasculature of the murine Kidney after segmentation. The poorly-resolved vessels in the renal corpuscles are displayed in yellow. Grid scale: 50µm. (**A’**) Detail of the same 3D-rendering. SB: 100µm.
4. Representative 3D-projection of the perfused vasculature in the murine liver after segmentation. Grid scale: 50µm.


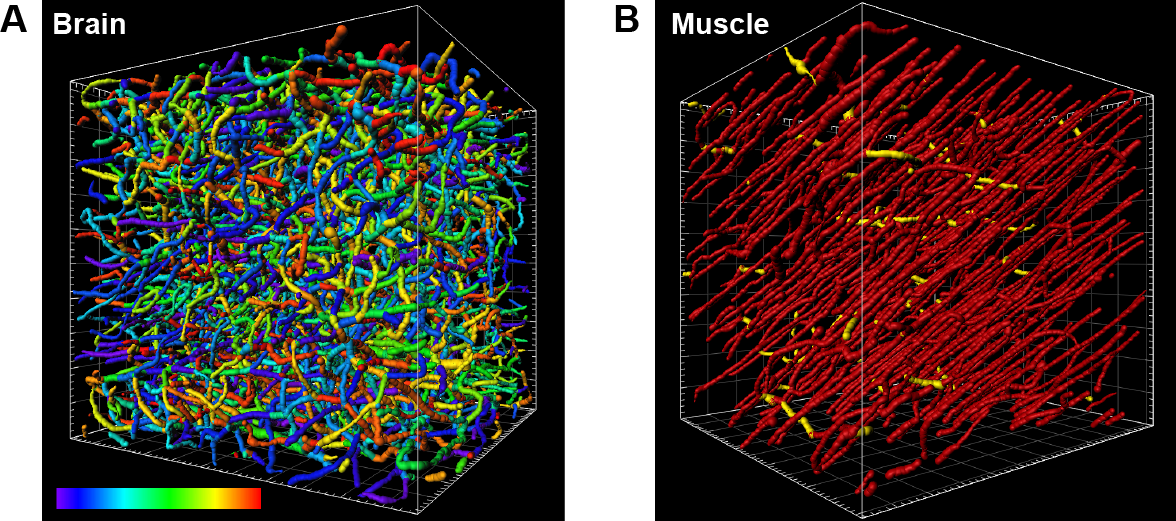


**Supplemental Figure S5: Orientation angel of vessels in the frontal cortex and the skeletal muscle**

1. Color-coded orientation angel of blood vessels in the murine brain (frontal cortex).
2. Orientation angel of blood vessels in the murine skeletal muscle. Vessels in or near the dominant orientation (0° ± 15°, along muscle fibers) are displayed in red, vessels perpendicular to this preferred orientation (90° ± 15°) in yellow.

Grid scale: 50µm.


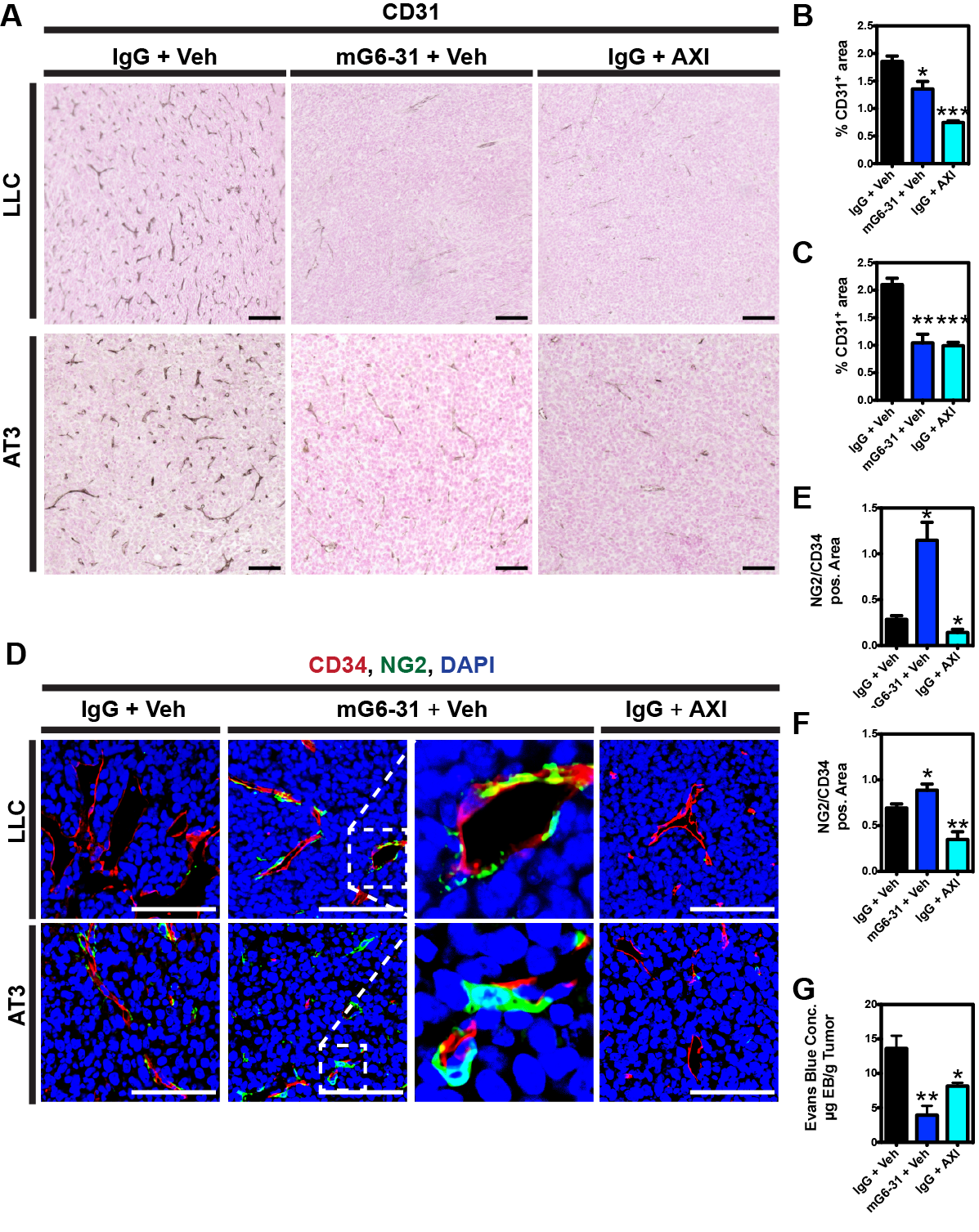


**Supplemental Figure S6: Effect of anti-angiogenic therapy on vessel density, maturation and patency.**

1. Section from LLC and AT3 tumors stained for the endothelial marker CD31 (Pecam) after treatment with either the mVEGF-A sequestering antibody mG6-31 or the TKI axitinib. In both tumor models treatment resulted in a reduced vessel density. SB: 100µm.
2. Quantification of CD31^+^ staining in LLC tumors after treatment with mG6-31 or axitinib.
3. Quantification of CD31^+^ staining in AT3 tumors after treatment with mG6-31 or axitinib.
4. Co-immunofluorescence staining for the endothelial marker CD34 (red) and the pericyte marker NG2 (CSPG4, green) of section from LLC and AT3 tumors after treatment with either mG6-31 or axitinib. In both tumor models treatment with mG6-31 resulted in improved pericyte coverage of capillaries, while axitinib further reduced vessel maturation.
5. Quantification of the ratio of NG2^+^/CD34^+^ staining in LLC tumors after treatment with mG6-31 or axitinib.
6. Quantification of the ratio of NG2^+^/CD34^+^ staining in AT3 tumors after treatment with mG6-31 or axitinib.
7. Quantification of extravasated Evan’s Blue in AT3 tumors after treatment with mG6-31 or axitinib. Both treatments improved vessel patency.

Error bars: ± SEM. Asterisks display results from statistical tests with *: P < 0.05, **: P < 0.01, ***: P < 0.001.


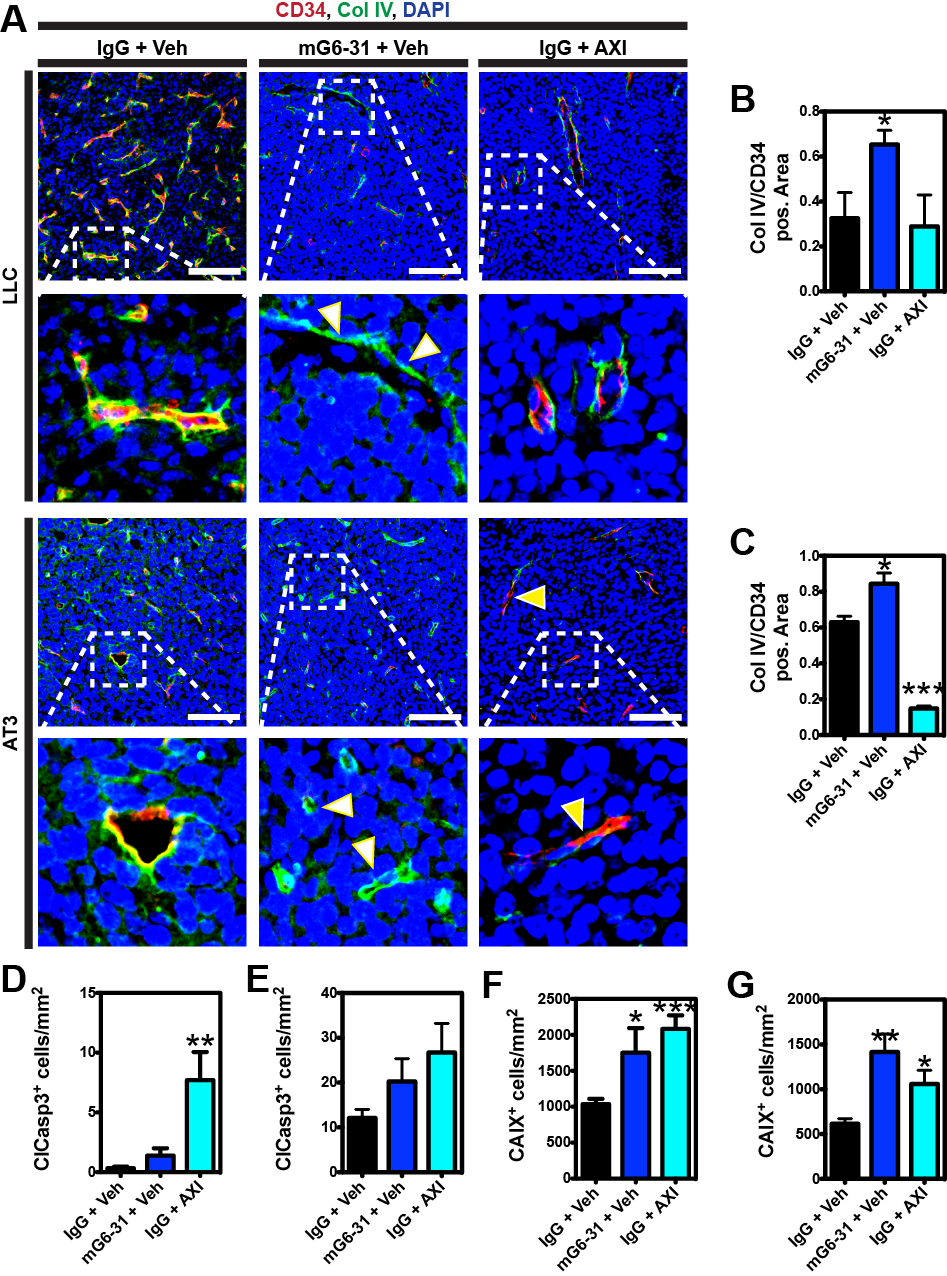


**Supplemental Figure S7: Effect of anti-angiogenic therapy on vessel pruning, apoptosis and tumor oxygenation.**

1. Co-immunofluorescence staining for the endothelial marker CD34 (red) and basal membrane-associated collagen IV (green) of section from LLC and AT3 tumors. After treatment with mG6-31 vessel density is reduced and empty collagen IV sleeves remain as leftover of pruned vessels (white arrow heads), Axitinib also reduced vessel density, but inhibited in parallel collagen IV production. This resulted in the AT3 model in an increased number of capillaries with absent collagen IV^+^ basal membrane (yellow arrow heads).
2. Quantification of the ratio of Col IV^+^/CD34^+^ staining in LLC tumors after treatment with mG6-31 or axitinib.
3. Quantification of the ratio of Col IV^+^/CD34^+^ staining in AT3 tumors after treatment with mG6-31 or axitinib.
4. Quantification of cells staining positive for the apoptosis marker cleaved caspase 3 in LLC tumors after treatment with mG6-31 or axitinib.
5. Quantification of cells staining positive for the apoptosis marker cleaved caspase 3 in AT3 tumors after treatment with mG6-31 or axitinib.
6. Quantification of cells staining positive for the hypoxia marker carbonic anhydrase IX in LLC tumors after treatment with mG6-31 or axitinib.
7. Quantification of cells staining positive for the hypoxia marker carbonic anhydrase IX in AT3 tumors after treatment with mG6-31 or axitinib.

Error bars: ± SEM. Asterisks display results from statistical tests with *: P < 0.05, **: P < 0.01, ***: P < 0.001.
